# Neutron Radiation Tolerance of Two Benchmark Thiophene-Based Conjugated Polymers: the Importance of Crystallinity for Organic Avionics

**DOI:** 10.1038/srep41013

**Published:** 2017-01-23

**Authors:** G. M. Paternò, V. Robbiano, K. J. Fraser, C. Frost, V. García Sakai, F. Cacialli

**Affiliations:** 1 London Centre for Nanotechnology, Department of Physics and Astronomy, University College London, Gower Street, London WC1E 6BT, UK; 2 ISIS Pulsed Neutron and Muon Source, Science and Technology Facilities Council, Rutherford Appleton Laboratory, Harwell Science and Innovation Campus, Didcot OX11 0QX, UK

## Abstract

Aviation and space applications can benefit significantly from lightweight organic electronics, now spanning from displays to logics, because of the vital importance of minimising payload (size and mass). It is thus crucial to assess the damage caused to such materials by cosmic rays and neutrons, which pose a variety of hazards through atomic displacements following neutron-nucleus collisions. Here we report the first study of the neutron radiation tolerance of two poly(thiophene)s-based organic semiconductors: poly(3-hexylthiophene-2,5-diyl), P3HT, and the liquid-crystalline poly(2,5-bis (3-tetradecylthiophen-2-yl)thieno[3,2-b]thiophene), PBTTT. We combine spectroscopic investigations with characterisation of intrinsic charge mobility to show that PBTTT exhibits significantly higher tolerance than P3HT. We explain this in terms of a superior chemical, structural and conformational stability of PBTTT, which can be ascribed to its higher crystallinity, in turn induced by a combination of molecular design features. Our approach can be used to develop design strategies for better neutron radiation-tolerant materials, thus paving the way for organic semiconductors to enter avionics and space applications.

π-conjugated polymers attract interest owing to their fundamental properties and applicability to devices spanning from organic light-emitting diodes (OLEDs)^1^,^2^ to thin-films transistors (OTFT)^3^,^4^ and photovoltaic diodes (OPVDs). Crucially, they combine the excellent mechanical characteristics of polymeric systems with the unique optoelectronic properties arising from the partly-delocalised π-orbitals, thus enabling fabrication of flexible, mechanically-robust, and lightweight optoelectronics^5^,^6^, with key advantages for those applications in which payload control is vital, such as avionic and, in perspective, space systems, which already heavily rely on the use of polymers as passive structural components^7^. Such benefits may also be exploited for smaller-scale applications, such as weather-balloons and mini-satellites, for which weight and volume requirements might be even more stringent than for spacecrafts, in spite of stability issues that have traditionally affected organic devices, but that can be appropriately be dealt with as clearly demonstrated by the commercialisation of organic TVs^8^. However, the influence of the relatively harsh radiation environments encountered by such devices needs to be better understood. For instance, it is estimated that equipment on the International Space Station (ISS) receive an annual fluence of ~2.8 × 10^11^ neutrons/cm^2^ (secondary neutrons generated by the interaction of cosmic rays with the Station vs. 3.85 × 10^5^ neutrons/cm^2^ at ground level), with energies from 10^−1^ to 10^11^ eV^9^.

Remarkably, there is no detailed study of the effect of neutron radiation on some of the most established organic semiconductors (OS), even though exposure to other types of radiation has been studied previously. For example, investigations of solar cells based on P3HT and [6,6]-phenyl-C61-butyric acid methyl ester (PCBM) showed degradation of all cell parameters upon exposure to X-rays which also depend on the PCBM loading, but interestingly degradation could not be associated to any significant change in the structure of the active layers^10^. Instead, it was attributed to the accumulation of excess charges generated by X-ray absorption at the electrodes. The latter can create a reverse electric field that reduces the built-in potential, in turn affecting the open circuit voltage, *V*_*oc*_. Street and collaborators^11^ noted instead the formation of deep-trap states in X-ray irradiated OPVDs that could act as recombination centres for charge carriers. More recently, the tolerance of pentacene organic field-effect transistors (FETs) to γ-rays has also been investigated^12^. By means of X-ray and ultraviolet photoelectron spectroscopies (XPS and UPS respectively), the authors observed increased *p*-doping and newly generated states that facilitated hopping transport.

Surprisingly, we found no studies on so-called “neutron radiation hardening” of organic devices, albeit neutrons representing one of the best tools to study the effect of cosmic rays and being an example of the most severe forms of radiation produced after nuclear interactions of cosmic rays with the structural shielding of spacecrafts^13^,^14^. Highly energetic neutrons cause atomic displacements and the generation of irreversible defects in materials, as well as reversible “soft-errors” (or “single-event effects”) both at sea^15^ and at flight level^16^. A significant neutron flux is also generated in the atmosphere by thunderstorms^17^. These neutrons, that are produced via a photonuclear reaction promoted by γ-rays born in the strong electric fields of thunderclouds, can pose significant hazards for civil aviation^18^. In general, it is thus crucial to shed light on the effects of neutron radiation with a broad energy spectrum, such as those available at a spallation source.

Here, we focus on two notable examples of conjugated polymers with significantly different degrees of crystallinity, namely: poly(3-hexylthiophene), P3HT^4^, and the liquid crystalline poly(2,5-bis(3-hexadecylthiophen-2-yl)thieno[3,2 b]thiophene), PBTTT^19^. In particular, we studied the effect of neutron irradiation on the optical, chemical and vibrational features of the polymers via ultraviolet-visible absorption (UV-Vis), XPS and Raman spectroscopy, and correlate these with the electrical properties as revealed by characterisation of FETs, incorporating these materials as active layers. We also thermally annealed the irradiated samples to investigate reversibility. We show in what follows that the two polymers behave rather differently, with PBTTT showing a greater radiation tolerance than P3HT. This can be correlated to the higher crystalline order and the more rigid and structurally stable backbone of PBTTT, in turn resulting from the presence of the thienothiophene moieties and from a lower density of solubilising chains.

## Results

**Figure 1:**
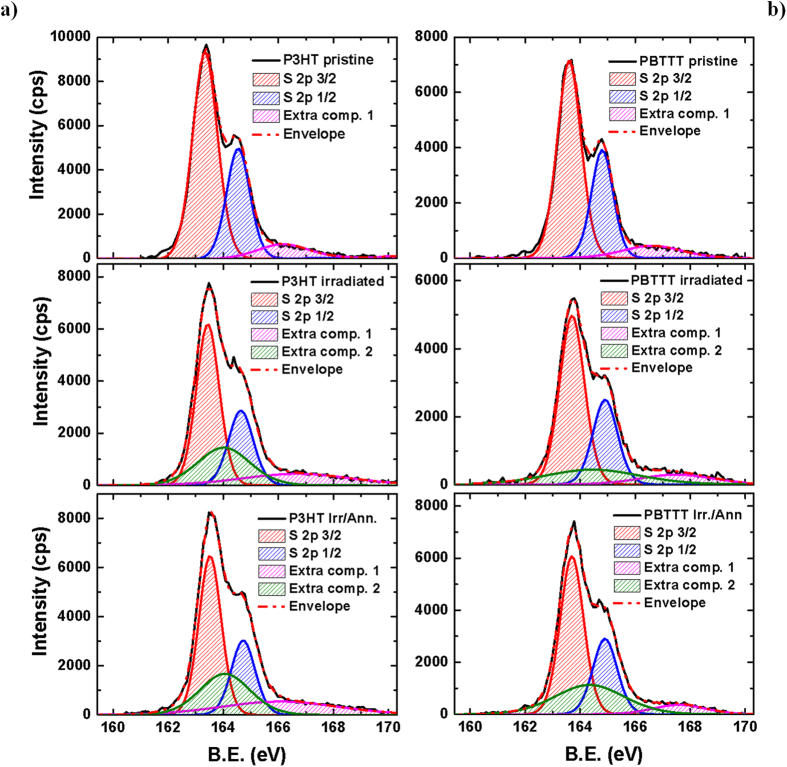
XPS Spectra. (**a**) XPS spectra (counts per second, cps vs. binding energy, B.E.) for pristine, irradiated and irradiated/annealed P3HT and PBTTT films (thickness ~80 nm) on silicon/silicon oxide substrates, alongside their fitting components. The XPS data were taken at a take-off angle of 90° for all the samples. The component centred at ~166 eV (purple line) is attributed to oxidative damage related to sample-handling, whereas the 164/164.5 eV component (green), can be ascribed to neutron-induced degradation, as absent from the spectra of the pristine films. The annealing was carried out 100 °C for 10 minutes in a nitrogen-filled glovebox.

First, we combined XPS and UV-Vis to quantify the impact of neutron radiation on the chemical and optical features of the macromolecules, and gain insight into the neutron-induced degradation mechanism. XPS spectra (and fits) of the S 2p peaks are shown in Fig. 1(a). The S 2p peak of “pristine” P3HT and PBTTT consists of two main components, namely S 2p_3/2_ and S 2p_1/2_, arising from the spin-orbit splitting in the p-type orbitals. We use “pristine” to indicate films that have not been yet irradiated, but that were subject to a 10-minute annealing at 100 °C after spin-coating. However, we also report results for films that have not been pre-annealed in the Electronics Supplementary Information, ESI. The area ratio between these two components was maintained in the fits at 2:1, to account for the degeneracy of each spin state, and we selected an energy separation of 1.2 eV, as described elsewhere^20^,^21^. Details of the procedure are presented in the ESI.

Neutron irradiation leads to significant and irreversible changes of both polymers, as can be inferred from the clear broadening of the S 2p lines, which alongside the chemical shift to higher binding energy (0.1 eV) has been linked to the presence of a positive charge on the sulfur resulting from oxidative doping^20^,^21^,^22^ of poly(thiophenes). Notably, thermal annealing after irradiation leads to a further shift of +0.1 eV for P3HT, but not for PBTTT.

From a quantitative point of view and starting with the pristine polymers, we note that the S 2p peak is fitted well with a 2p_3/2_/2p_1/2_ doublet, plus a small extra-component at higher binding energies (namely 166.2 eV and 166.6 eV for P3HT and PBTTT, respectively) that might account for a slight oxidation of the surface, owing to unavoidable exposure to oxygen and moisture during handling (see ESI for XPS survey spectra – Supplementary Fig. S1 - and elements concentration). In contrast, we need an additional component to fit the S 2p line of the same films after irradiation (164 eV for P3HT and 164.5 eV for PBTTT). We note two further remarkable features here: first, the total area of the extra components is higher for P3HT than for PBTTT (i.e. the sum of the components at ~166 eV and 164 eV are ~36.8% and 24.8% of the total area of the features between 160 and 170 eV, respectively), and second, it keeps growing upon annealing (to 40.6% and 31.4% for P3HT and PBTTT, respectively), thus indicating that the population of such species is enhanced, especially for PBTTT. Although, such a feature has been linked to the presence of polaronic species in oxidised poly(thiophenes)^20^,^21^, XPS survey spectra (ESI) do not correlate with the trend of the S 2p line, but indicate a reduction of oxygen upon annealing, thereby strongly suggesting that the features at 164 eV and 164.5 eV, is mainly a neutron-induced effect.

**Figure 2:**
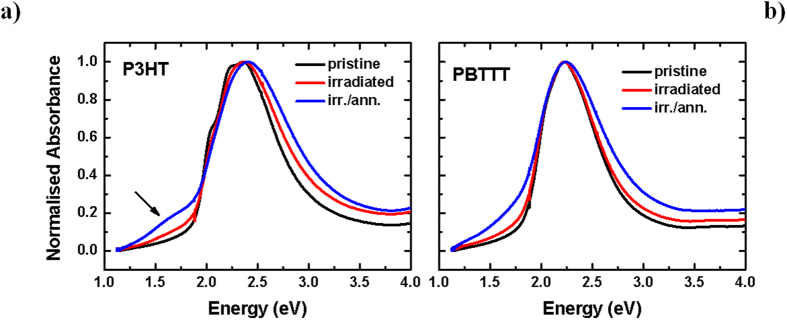
Absorption Spectra. (**a**) Normalised UV-VIS absorption spectra (uncorrected for reflection) for P3HT and (**b**) PBTTT pristine, irradiated and irradiated/annealed films (~80 nm, on spectrosil substrates, at room temperature). The annealing was carried out at 100 °C for 10 minutes in a nitrogen glove-box.

We also find indications of increased energetic disorder in the UV-VIS spectra of P3HT and PBTTT (Fig. 2). In fact, we observe loss of the vibronic progression on the low-energy side of the first absorption peak (~555 nm and 605 nm, respectively) that is commonly associated with a high degree of inter-molecular order in regioregular P3HT^23^,^24^,^25^. A slight blue-shift (~0.1 eV, ~11 nm) of the main absorption feature accompanies a bleaching of the π-π* transition, and can be accounted for by both disruption of conjugation and inter-chain order. An irradiation-induced increase of the “sub-gap” optical absorption, taken together with the XPS data above, suggests a transition from neutral to oxidised (polaronic/bipolaronic) states in poly(thiophene)^26^,^27^ and the concomitant formation of defects in the gap. Also in good agreement with the XPS data, these changes are more prominent when a thermal treatment is carried out on the irradiated films, as we observe a further blue-shift (~0.1 eV) and even stronger sub-gap absorption, so prominent for P3HT (despite the slight recovery of the bleaching, see Supplementary Fig. S2 in the ESI), to suggest development of an additional peak in the low-energy region (~1.6 eV). We note that it is not possible to explain the feature on the low-energy side of the P3HT main absorption band (for the irradiated/annealed sample, see arrow, Fig. 2a) on the basis of scattering alone, as also confirmed by inspection of the first derivative of the spectrum (not shown) in which a peak is unequivocally identifiable. Remarkably, both blue-shift and growth of sub-gap tail are significantly less pronounced for PBTTT compared to P3HT (despite very similar neutron scattering cross-sections), and especially before the post-irradiation annealing.

We now turn to Raman spectroscopy to obtain a deeper insight into the neutron-induced modifications of the two polymer films since Raman and more generally vibrational spectroscopies have been used effectively to assess fundamental structure-property relationships of polyconjugated systems^28^,^29^.

**Figure 3:**
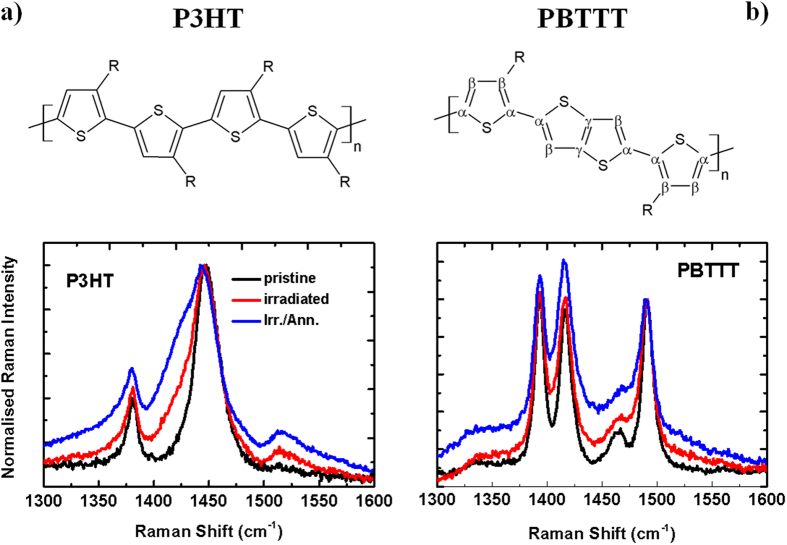
Raman Spectra. Raman spectra for P3HT (left) and PBTTT (right) thin films (~85 nm) on spectrosil substrates for excitation with a diode laser at 785 nm, at room temperature. All P3HT and PBTTT spectra are normalised to the peak intensity at 1445 cm^−1^ and 1490 cm^−1^, respectively.

We report the full spectra in the 600–1600 cm^−1^ region (already previously assigned^30^,^31^) in the ESI (Supplementary Fig. S3), and focus instead in Fig. 3 on the 1300–1600 cm^−1^ region. This is particularly interesting because it includes the two main in-plane ring modes: the C=C symmetric stretching at 1445 cm^−1^, and the C-C intra-ring stretching at 1380 cm^−1^, that are known to be sensitive to π-electron delocalisation^32^ and degree of structural order^33^ in poly(thiophenes).

Starting first with P3HT, we can summarise the effects of neutron irradiation as follows:

- A general broadening of the spectral features in the 1300–1600 cm^−1^ region for the irradiated samples. Such a broadening increases further after thermal annealing of the irradiated sample, indicating lack of recovery and actually further degradation induced by the treatment. A similar broadening has previously been ascribed to a decrease of the degree of molecular order and conjugation length in poly(thiophene) films^33^,^34^.
- A new low-energy wing of the C=C symmetric stretching peak appears at 1430 cm^−1^ after irradiation. This contribution has been previously assigned to the symmetric C=C stretching mode of the quinoid units of oxidised P3HT^35^. Appearance of the 1430 cm^−1^ band has also been observed when P3HT is exposed to the combined action of UV light and oxygen^36^, and linked to an order/disorder transition taking place upon oxidation of P3HT chains to their quinoid form^30^. In full agreement with the previously mentioned trend, thermal annealing enhances such a wing, instead of favouring recovery to the pristine form, thus amplifying and possibly consolidating the neutron-induced damage in the polymer (see Supplementary Fig. S4 in the ESI for the fits of Raman peaks in the 1300–1600 cm^−1^ region).
- The appearance of the anti-symmetric C=C stretching peak at 1515 cm^−1^ and the concomitant enhancement in the C-C inter-ring peak at 1210 cm^−1^ and the C-S-C ring deformation peak at 728 cm^−1^ (see Supplementary Fig. S3 for the full spectrum). We assign these to the formation of non-coplanar segments in the chains in agreement with previous literature that used chemical doping to drive the formation of polaronic species^30^,^35^,^37^, which are expected to induce torsional disorder along the polymer backbone, consistent with the suppression of the rigidity and conjugation of the thiophenes.

Let us now turn to the Raman spectra of PBTTT (Fig. 3b) and focus first on the C=C-C ring stretch region at 1350–1550 cm^−1^ for the same reasons illustrated above for P3HT^32^ (for completeness we report the full spectrum in the Supplementary Information). We observe four strong peaks at 1393, 1418, 1463, and 1493 cm^−1^. These correspond to C_β_-C_β_ intra-ring stretch, C_γ_-C_γ_ intra-ring stretch, C_α_-C_α_ inter-ring stretch and C_α_-C_β_ intra-ring stretch, respectively^38^ (please see Fig. 3 for the labelling convention of the different carbons). We note a general broadening of the spectra in this region, with the C_α_-C_α_ inter-ring stretch peak at 1463 cm^−1^ predominantly affected by the post-radiation thermal annealing. This may indicate an increased conformational disorder of the backbone upon irradiation^38^, even though the polymer largely retains its overall conformational order, as suggested by the minimal changes occurring in the other in-plane backbone modes and in the C-C inter-ring peak at 1210 cm^−1^, in the C-H bending at 1084 cm^−1^, and in the C-S-C ring deformation mode at 700 cm^−1^.

To summarise, the Raman data agree well with the XPS and optical absorption, consistent with a neutron-induced doping process as the main degradation pathway for irradiated P3HT and PBTTT, with PBTTT exhibiting a higher radiation tolerance.

### OFETs characteristics

The optical and chemical changes observed suggest that PBTTT is intrinsically more resilient than P3HT following irradiation with neutrons and provide a first indication of the material design criteria to be followed to increase radiation hardness of conjugated polymers. However, a more direct and quantitative evaluation of the mobility and overall transport properties is needed for applications such as FETs and more generally logic circuits. We therefore investigated the influence of neutron irradiation on FETs incorporating P3HT and PBTTT. In addition, irradiated devices were thermally annealed to establish whether radiation-induced effects are reversible.

**Figure 4:**
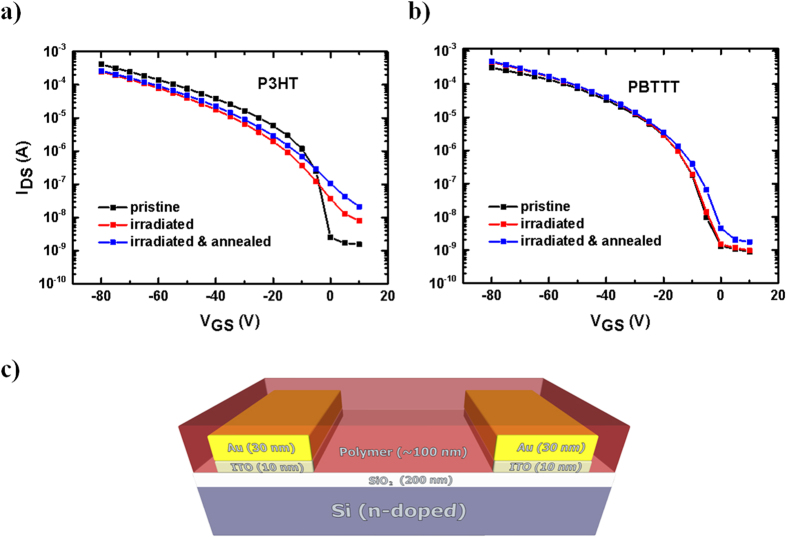
Field-Effect Transistors (FETs). (**a**) OFET transfer characteristics of P3HT and (**b**) PBTTT (y-axis in logarithmic scale) taken at V_DS_ = −80; (**c**) sketch of bottom gate/ bottom contact OFET architecture. Note that these curves are taken on the same device under the three experimental conditions (pristine, irradiated and irr./ann.), and are representative of the electric behaviour of six devices.

Figures 4a and b show the transfer characteristics for P3HT and PBTTT respectively, taken at V_DS_ = −80 V and V_GS_ from 0 to −80 V. In P3HT-based devices the current at high - voltage and the mobility slightly decrease after irradiation but, if thermally annealed after the irradiation step, the mobility is almost recovered to ~92% of the initial value (Table 1). On the other hand, the threshold voltage *V*_*T*_ (Table 2), as well as the OFF current (Table 3) and the sub-threshold slope (Table 4) increase when going from pristine to irradiated and irradiated/annealed devices. In this context, the increase in I_DS_ at low-voltage and in the sub-threshold region upon irradiation, as well as the partial recovery of the mobility after further annealing appear to be triggered by the formation of neutron-induced defective species in the channel^39^, consistent with oxidative doping. As a result, the extraction of the hole-mobility values is influenced heavily by the presence of such defective states, especially in the low-voltage regime.

In stark contrast to P3HT devices, PBTTT-based ones only display a marginal increase of mobility, *V*_*T*_ and sub-threshold parameters (OFF current and sub-threshold slope) even after post-radiation annealing. Therefore, the electrical characteristics correlate remarkably well with the optical, XPS and Raman results, providing clear corroborating evidence of a significantly higher tolerance to neutron radiation of PBTTT vs. P3HT.

## Discussion

The combination of chemical, optical, vibrational and electrical characterisations suggests that neutron irradiation leads to oxidative-like doping of the materials. In addition, our findings indicate that the population of such doped species and the subsequent decrease of conformational order in both polymers is amplified by the post-irradiation thermal annealing. However, although the degradation mechanism appears to be in principle similar for P3HT and PBTTT, the two polymers show a different tolerance, with PBTTT being more resilient than P3HT. Disruption of the chemical and electronic order upon irradiation and post-irradiation annealing also deteriorates the switching ability of P3HT devices affected by a steep drop of the ON/OFF ratio, consistent with a neutron-induced doping in the material. Hole-mobility decreases after irradiation, although it recovers partially (92%) to its initial value if the device is thermally annealed. This is surprising, as one would expect that a higher amount of doped chains would lead to an increase of the charge mobility. In fact, this regime of hole-mobility has been observed by Neher and co-workers for chemically-doped P3HT^40^. They noticed a mobility decrease at low-to-medium doping levels, for which the beneficial effect of doping is compensated by the deleterious increase of energetic disorder, but an increase at sufficiently-high doping levels. We propose that the same phenomenon also occurs in our system, in which the thermal annealing drives the transition from a low to a moderate doping regime, thus enabling partial recovery of the hole-mobility.

**Figure 5:**
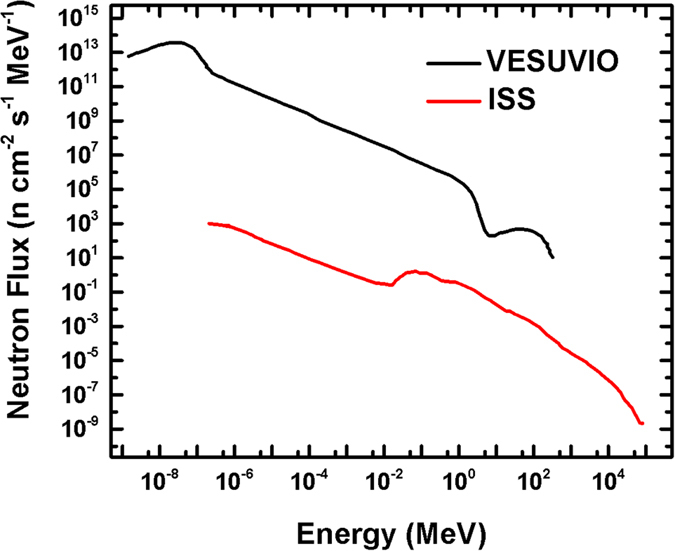
Neutrons Energy Spectra. Experimental differential neutron energy spectrum at VESUVIO^41^ vs. estimated differential energy spectrum received by the ISS^9^. Adapted from Fig. 3 of reference^9^ (Armstrong TW, Colborn BL. Predictions of secondary neutrons and their importance to radiation effects inside the international space station. *Radiat. Meas.* 33, 229-234 (2001), doi:10.1016/S1350-4487(00)00152-9), and from Fig. 4 of reference^41^ (Bedogni R*, et al*. Characterization of the neutron field at the ISIS-VESUVIO facility by means of a bonner sphere spectrometer. *Nucl. Instrum. Methods Phys. Res., Sect. A* 612, 143–148 (2009), doi:10.1016/j.nima.2009.09.004).

Turning to the actual neutron-induced degradation mechanism, we first consider the experimental radiation environment. Comparison between the experimental neutron energy spectrum at the VESUVIO beamline^41^ and the calculated energy spectrum of the neutrons generated on board the ISS^9^ as a result of its interaction with cosmic radiation is shown in Fig. 5. In both cases, the energy spectrum is extremely broad, ranging from cold to ultrafast neutrons. This may lead to a plethora of different neutron-nucleus events, depending on the neutron energy and nuclei properties (i.e. absorption and scattering cross-sections). However, we can clearly observe that the VESUVIO spectrum is significantly more intense in all the regions, with the ISS spectrum being seven orders of magnitude less intense for epithermal-neutrons (10^−6^–10^−1^ MeV). Our experiment is thus an accelerated irradiation test, which enables mimicking several years of space irradiation in only a few hours. E.g. focusing on neutrons with energy >10 MeV, for which the nominal value for the VESUVIO flux is 5.82 × 10^4^ n cm^−2^ s^−1^, we estimate that in 4.5 hrs our samples received the equivalent of ~45 years of irradiation on the ISS. It is also worth reporting that a γ-ray background, with energy lines ranging from hundreds to thousands keV, is also present in our experimental blockhouse^42^,^43^,^44^. Such background of energetic photons arises from neutron capture, absorption and inelastic scattering events given by the element present in the experimental set-up and in the wall of the blockhouse. Unfortunately, a quantitative calculation of the fluence magnitude of this contribution was not available at the time of writing, and, therefore, we cannot estimate to what extent γ-rays would interact effectively with our samples and eventually lead to degradation effects.

Neutron-induced damage can be classified as due to either ionisations by nuclear reactions/collisions, or to recoil, resonances and cleavage events. The former originate from direct neutron-nucleus collisions and involve mainly fast neutrons (>0.1 MeV), whereas the latter can be attributed to the interaction of epithermal neutrons with the nuclei. If the deposited energy is large enough to break a C-C or a C-H bond (~4 eV), cleavage phenomena will follow. We consider that such events are likely to be responsible for the damage observed in our experiments because of the synergistic effects of the higher fluence of epithermal neutrons in our experiment (2 × 10^15^ neutrons cm^−2^) than fast (>0.1 MeV) neutrons (7.8 × 10^8^ cm^−2^), and the high probability of interactions with epithermal neutrons (either by scattering or absorption) of such highly hydrogenated systems. The relatively strong tendency of conjugated polymers and of organic matter in general to scatter neutrons stems in fact from their significant content of hydrogen atoms, which exhibit the highest scattering cross section among the isotopes^45^. While we cannot completely rule out the presence of neutron-induced damage in the substrate (i.e. in the gate oxide), we did not observe any increase of the gate leakage current upon irradiation, thus suggesting most damage is within the highly-hydrogenated active layer.

We thus propose that neutron-induced degradation of these polymers is a radical mechanism likely activated by hydrogen or alkyl abstraction, as for X-rays^11^ and UV-irradiated P3HT^46^, but activated in our case by either direct neutron-nucleus collisions (fast neutrons) or by inelastic processes (epithermal neutron/hydrogen interactions in the majority of cases), rather than by photo-generated electrons as for X-rays and UV (quantitative estimation of the rate of such events and calculation of deposited energy in the sample would require further computational analysis, which is beyond the scope of this manuscript).

Furthermore, it is useful to compare X-ray diffraction (XRD) spectra taken before and after the irradiation to extract further information on the influence of neutron irradiation on the crystalline fractions of the materials. Such XRD spectra do not show significant differences (Supplementary Fig. S5) thereby indicating that neutron-induced structural damage on the crystalline phase of either polymer is either absent or below the XRD detection threshold. Such an observation powerfully correlates with the differences we noted already between P3HT and PBTTT and leads us to propose that the degradation process mainly occurs in the amorphous phase, in which the defective species (i.e. radicals) would also have a higher mobility and reactivity^47^. As an additional, concurrent factor, we note that crystalline regions might also act as traps for the radicals and hence slow down the degradation process, as previously observed for γ-irradiated cellulose^48^ and carbohydrates^49^. In this scenario, thermal annealing of the irradiated films facilitates de-trapping of the radicals and thus exacerbates degradation, as indeed observed as the reduction of the P3HT transistors ON/OFF ratio. So we can explain the different degradation pathways of the two polymers in terms of a higher crystallinity of PBTTT. Effectively, a significant fraction of the neutron-induced defects are frozen at room temperature within the more extended crystalline phase, but with the possibility of being thermally activated. The prominent role played by the crystalline phase in (inhibiting) damage is further confirmed by the properties of non-annealed films (Experimental SI), in which the crystalline fraction is significantly lower than in the annealed ones. Remarkably, these demonstrate significantly lower tolerance for both non-annealed P3HT and PBTTT (OFETs measurements, Supplementary Fig. S6 and Table ST3), with the drain current and hole-mobility increasing after irradiation for both polymers and before thermal annealing. This is in full agreement with the expectation of a higher concentration of radicals in the amorphous phase (now more extended that in the pre-annealed case), i.e. not “frozen” within the crystalline fraction, that do not need a post-irradiation annealing to be ‘activated’.

We thus attribute PBTTT’s enhanced neutron radiation tolerance to a significantly more crystalline structure. Interestingly, this polymer was intentionally designed to assemble into large crystalline domains from a liquid-crystal phase and exhibits a planar and rigid π-conjugated system, which eventually leads to high charge mobility (~1 cm^2^ V^−1^ s^−1^)^19^. It has been even suggested that in this material the chain interaction and ordering is favoured already in solution^50^ and is preserved during the crystallization. Key guiding principles of such molecular design are: (i) the introduction of the linear conjugated co-monomer, thieno[3,2-b]thiophene, which facilitates the adoption of the low-energy backbone conformation and hence provides more conformational backbone stability; (ii) the low side-chain attachment density (in fact half than in P3HT), that permits interdigitation between the chains and the formation of stable and large three-dimensional crystalline domains^51^.

In conclusion, we have investigated, for the first time, the neutron radiation tolerance of two important polymeric semiconductors, namely P3HT and PBTTT. Neutrons represent one of the most disruptive components in cosmic-ray particle cascades, and pose a number of hazards to the electronic equipment in aircrafts, spacecrafts and orbiting objects. Evidence we collected through a variety of spectroscopic (XPS, UV-Vis, PL, Raman) and device characterisation (FETs) strongly suggests that neutron irradiation promotes the polaronic species in both these polymers. Remarkably, degradation is much less pronounced in PBTTT, indicating a superior radiation tolerance of this polymer. We propose that the main degradation process is a radical, non-oxygen assisted mechanism, that can be rationalised as a “radiation-induced doping” taking place preferentially in the amorphous phase of the thin films. Compared to P3HT, PBTTT thus features superior radiation tolerance primarily as a result of its superior crystalline order. The results above provide molecular design guidelines for polymeric semiconductors with improved neutron resilience, and pave the way towards the effective utilisation of such polymer-based electronics for space and avionics applications.

## Materials and Methods

### OFETs fabrication and characterisation

Regioregular P3HT (Aldrich) and C16-PBTTT (Ossila) were used as received, without further purification. P3HT and PBTTT solutions were prepared by dissolving 10 mg/mL in chlorobenzene at room temperature (RT) and in 1, 2 dichlorobenzene at 80 °C respectively. For the fabrication of bottom-gate/bottom-contact OFETs (schematic in Fig. 4b), we started from n-doped silicon substrates with a 230 nm SiO_2_ layer, patterned with interdigitated indium tin oxide, ITO, (10 nm)/Au (30 nm) source and drain contacts (channel length, L = 5–20 μm and channel width, Z = 1 cm, purchased from Fraunhofer IPMS, Dresden, Germany). The substrates were sonicated in acetone and isopropyl alcohol for 10 minutes, dried in a flux of dry nitrogen and then placed in an oxygen-plasma asher for another 10 minutes to increase the substrate wettability and the Au electrodes work function^25^. A hexamethyldisilazane (HMDS) layer was spin-coated on the samples, annealed at 100 °C for 1 h and spin-rinsed with isopropyl alcohol. Eventually, the active films were deposited by spin-casting the polymer solutions onto the transistor substrates (1500 rpm for 60 seconds). As PBTTT is soluble in chlorinated solvents only above 70 °C, to prevent the formation of a defect-rich interface due to the quick crystallization of the solution upon contacting the cooler substrate, both the PBTTT solution and the substrates were kept at the same temperature (~70 °C) before spin-coating. All devices except those of Supplementary Fig. S6 and Supplementary Table ST3 were thermally annealed at 100 °C for 10 minutes as it is usual to improve the transistor performance. Therefore, with the term “pristine” we refer to devices that have not yet been exposed to neutrons, but that have been thermally annealed before such exposure. To look at the effect of the thermal “pre-annealing” we have also irradiated some devices that were not annealed soon after spin-coating, and report their properties in Supplementary Fig. S6 and Supplementary Table ST3 of the ESI.

The characteristics of the OFETs were measured using a Karl Suss PM5 probe station and a HP4145 parameter analyser, which was connected to low-noise guarded probes for the source and drain-contacts and to the probe chuck for the gate connection. The HMDS/active-layer deposition and electrical characterization of the OFETs was carried out in a dry box under a nitrogen atmosphere with less than 20 ppm residual water vapour and 5 ppm oxygen.

The reported mobilities were measured in the saturation regime (at −80 V) by taking the slope of the square root of the I_DS_ plotted against the V_GS_ and fitted to the saturation regime equation (1),


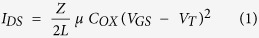


where Z and L are channel width and length respectively, μ the field-effect mobility, C_ox_ the capacitance of the gate dielectric per unit area, V_GS_ the gate voltage and V_T_ the threshold voltage. All the parameters reported in this manuscript represent the average over six measurements performed on six different devices. Note that, given the little amount of time available to perform the experiment at the large scale facility, we did not attempt to optimise the OFETs performances (i.e. active layer thickness, choice of passivation layer and annealing temperature/time). However, we think that the large difference in terms of crystallinity between P3HT and PBTTT (see for example Supplementary Fig. S5), allows us to make a significant comparison between these two polymers, even if the experimental conditions were not fully optimised.

### Irradiation with high-energy neutrons

To test the tolerance of P3HT and PBTTT to neutron radiation, we used the highly energetic flux of the VESUVIO beam line (in the range of MeV) at the ISIS Neutron and Muon Facility, (UK). The neutron production at ISIS relies upon spallation reactions induced by 800 MeV proton bunches accelerated in a synchrotron. The energy distribution of the neutron in the beam ranges from cold (0.025 eV) to fast (>10 MeV) and ultrafast (>20 MeV) neutrons. At the normal primary proton current (180 μA.hrs) the integrated neutron flux above 10 MeV was determined to be 5.82 × 10^4^ · n · cm^−2^ · s^−1 ^52,53. In our experiment, we irradiated each sample with a total 7.8 × 10^8^ neutrons.cm^−2^ with energies higher than 0.1 MeV and 2 × 10^15^ neutrons.cm^−2^ for neutrons with energies from 1 eV to 0.1 MeV (irradiation time = 4.5 hrs). All the neutron irradiation process was carried out in air.

### X-ray photoelectron spectroscopy

XPS spectra were acquired using a Thermo Scientific K-Alpha photoelectron spectrometer with a monochromatic Al Ka source (1486.6 eV) and spectral intensity >2.5 Mcps at FWHM 1.0 eV on Ag 3d 5/2. The fitting procedure was carried out by using CasaXPS software.

### UV-Visible and Raman measurements

For the optical measurements, P3HT and PBTTT solutions were spin cast onto fused silica substrates (Spectrosil®). UV-Visible absorption measurements were done with a photo-spectrometer (Agilent 8453). Raman spectra were collected using a Renishaw inVia Raman microscope (50× objective) with an excitation wavelength of 785 nm (non-resonant conditions). The laser power was set at 1% of its maximum power to avoid photo-degradation of the sample (1–5 mW). The spectra were taken at RT in the Stokes’ region and were calibrated against the 520.5 cm^−1^ line of an internal silicon wafer. The signal-to-noise ratio was enhanced by repeated acquisitions. All spectra were corrected to subtract the samples fluorescence background.

## Additional Information

**How to cite this article**: Paternò, G. M. *et al*. Neutron Radiation Tolerance of Two Benchmark Thiophene-Based Conjugated Polymers: the Importance of Crystallinity for Organic Avionics. *Sci. Rep.* **7**, 41013; doi: 10.1038/srep41013 (2017).

**Publisher's note:** Springer Nature remains neutral with regard to jurisdictional claims in published maps and institutional affiliations.

## Supplementary Material

Supplementary Information

## Figures and Tables

**Table 1:** (a) FETs mobilities calculated in the saturation regime (V_DS_ = −80 V and V_GS_ between 0 and −80 V) by using eq. 1.

	Mobility pristine (cm^2^ V^−1^ s^−1^)	Mobility irradiated (cm^2^ V^−1^ s^−1^)	Mobility irr./ann (cm^2^ V^−1^ s^−1^)
P3HT	0.012 (4)	0.004 (1)	0.011 (1)
PBTTT	0.044 (1)	0.044 (1)	0.046 (1)

The numbers in parentheses represent the uncertainty of the averaged taken over six different devices.

**Table 2:** Threshold voltage (V_T_) for pristine, irradiated and irradiated/annealed P3HT and PBTTT transistors.

	V_T_ pristine (V)	V_T_ irradiated (V)	V_T_ irr./ann (V)
P3HT	−16.8 (8)	−21 (2)	−18 (2)
PBTTT	−13 (1)	−19 (5)	−20 (3)

The numbers in parentheses represent the uncertainty of the averaged taken over six different devices.

**Table 3:** ON/OFF ratio for pristine, irradiated and irradiated/annealed P3HT and PBTTT transistors.

	ON/OFF ratio × 10^6^ pristine	ON/OFF ratio × 10^6^ irradiated	ON/OFF ratio × 10^6^ irr./ann.
P3HT	0.3 (2)	0.027 (5)	0.0095 (9)
PBTTT	3.9 (3)	1.9 (6)	1.1 (4)

The numbers in parentheses represent the uncertainty of the averaged taken over six different devices.

**Table 4:** Sub-threshold slope and values for pristine, irradiated and irradiated/annealed P3HT and PBTTT transistors.

	Sub-thr. Slope pristine (V/decade)	Sub-thr. Slope irradiated (V/decade)	Sub-thr. Slope Irradiated/annealed (V/decade)
P3HT	3 (1)	10 (2)	11.5 (3)
PBTTT	2.3 (6)	2.9 (5)	3.6 (2)

The numbers in parentheses represent the uncertainty of the averaged taken over six different devices.

## References

[b1] Burroughes J. H. . Light-emitting diodes based on conjugated polymers. Nature 347, 539–541 (1990).

[b2] Tang C. W. & VanSlyke S. A. Organic electroluminescent diodes. Appl. Phys. Lett. 51, 913–915 (1987).

[b3] Bao Z., Dodabalapur A. & Lovinger A. J. Soluble and processable regioregular poly(3-hexylthiophene) for thin film field-effect transistor applications with high mobility. Appl.Phys.Lett. 69, 4108–4110 (1996).

[b4] Sirringhaus H., Tessler N. & Friend R. H. Integrated optoelectronic devices based on conjugated polymers. Science 280, 1741–1744 (1998). 9624049 10.1126/science.280.5370.1741

[b5] Kaltenbrunner M. . Ultrathin and lightweight organic solar cells with high flexibility. Nat. Commun. 3, 770, 10.1038/ncomms1772 (2012). 22473014 PMC3337988

[b6] Yi H. T., Payne M. M., Anthony J. E. & Podzorov V. Ultra-flexible solution-processed organic field-effect transistors. Nat. Commun. 3, 1259, 10.1038/ncomms2263 (2012). 23232389

[b7] Grossman E. & Gouzman I. Space environment effects on polymers in low earth orbit. Nucl. Instrum. Meth. B 208, 48–57 (2003).

[b8] Flatpanelshd http://www.flatpanelshd.com/news.php?subaction=showfull&id=1465304750 (2016) (accessed 07/11/2016).

[b9] Armstrong T. W. & Colborn B. L. Predictions of secondary neutrons and their importance to radiation effects inside the international space station. Radiat. Meas. 33, 229–234 (2001). 10.1016/s1350-4487(00)00152-9 11852942

[b10] Kumar A., Devine R., Mayberry C., Lei B., Li G. & Yang Y. Origin of Radiation-Induced Degradation in Polymer Solar Cells. Adv. Funct. Mater. 20, 2729–2736 (2010).

[b11] Street R. A., Northrup J. E. & Krusor B. S. Radiation induced recombination centers in organic solar cells. Phys. Rev. B 85, 205211–205224 (2012).

[b12] Raval H. N., Sutar D. S., Nair P. R. & Ramgopal Rao V. Investigation of effects of ionizing radiation exposure on material properties of organic semiconducting oligomer – Pentacene. Org. Electron. 14, 1467–1476 (2013).

[b13] Yajima K. . Measurements of Cosmic-Ray Neutron Energy Spectra from Thermal to 15 MeV with Bonner Ball Neutron Detector in Aircraft. J. Nucl. Sci. Technol. 47, 31–39 (2010).

[b14] Koshiishi H., Matsumoto H., Chishiki A., Goka T. & Omodaka T. Evaluation of the neutron radiation environment inside the International Space Station based on the Bonner Ball Neutron Detector experiment. Radiat. Meas. 42, 1510–1520 (2007).

[b15] Ziegler J. F. & Lanford W. A. The Effect of Sea-Level Cosmic-Rays on Electronic Devices. J. Appl. Phys. 52, 4305–4312 (1981).

[b16] O’Gorman T. J. . Field testing for cosmic ray soft errors in semiconductor memories. IBM J. Res. Dev. 40, 41–50 (1996).

[b17] Shah G. N., Razdan H., Bhat C. L. & Ali Q. M. Neutron generation in lightning bolts. Nature 313, 773–775 (1985).

[b18] Drozdov A., Grigoriev A. & Malyshkin Y. Assessment of thunderstorm neutron radiation environment at altitudes of aviation flights. J. Geophys. Res-Space 118, 947–955 (2013).

[b19] McCulloch I. . Liquid-crystalline semiconducting polymers with high charge-carrier mobility. Nat. Mater. 5, 328–333 (2006). 16547518 10.1038/nmat1612

[b20] Jenkins J. L., Lee P. A., Nebesny K. W. & Ratcliff E. L. Systematic electrochemical oxidative doping of P3HT to probe interfacial charge transfer across polymer–fullerene interfaces. J. Mater. Chem. A 2, 19221–19231 (2014).

[b21] Ratcliff E. L., Jenkins J. L., Nebesny K. & Armstrong N. R. Electrodeposited, “Textured” Poly(3-hexyl-thiophene) (e-P3HT) Films for Photovoltaic Applications. Chem. Mater. 20, 5796–5806 (2008).

[b22] Naudin E., Dabo P., Guay D. & Belanger D. X-ray photoelectron spectroscopy studies of the electrochemically n-doped state of a conducting polymer. Synth. Met. 132, 71–79 (2002).

[b23] Clark J., Silva C., Friend R. H. & Spano F. C. Role of intermolecular coupling in the photophysics of disordered organic semiconductors: aggregate emission in regioregular polythiophene. Phys. Rev. Lett. 98, 206406–206409 (2007). 17677723 10.1103/PhysRevLett.98.206406

[b24] Li G. . High-efficiency solution processable polymer photovoltaic cells by self-organization of polymer blends. Nat. Mater. 4, 864–868 (2005).

[b25] Seidler N., Lazzerini G. M., Li Destri G., Marletta G. & Cacialli F. Enhanced crystallinity and film retention of P3HT thin-films for efficient organic solar cells by use of preformed nanofibers in solution. J. Mater. Chem. C 1, 7748–7757 (2013).

[b26] Chung T. C., Kaufman J. H., Heeger A. J. & Wudl F. Charge Storage in Doped Poly (Thiophene) - Optical and Electrochemical Studies. Phys. Rev. B 30, 702–710 (1984).

[b27] Skompska M. & Szkurlat A. The influence of the structural defects and microscopic aggregation of Poly(3-alkylthiophenes) on electrochemical and optical properties of the polymer films: discussion of an origin of redox peaks in the cyclic voltammograms. Electrochim. Acta 46, 4007–4015 (2001).

[b28] Castiglioni C., Delzoppo M. & Zerbi G. Vibrational Raman-Spectroscopy of Polyconjugated Organic Oligomers and Polymers. J. Raman Spectrosc. 24, 485–494 (1993).

[b29] Zerbi G. Vibrational Spectroscopy of Conducting Polymers: Theory and Perspective. In: Handbook of Vibrational Spectroscopy. John Wiley & Sons, Ltd (2006).

[b30] Baibarac M., Lapkowski M., Pron A., Lefrant S. & Baltog I. SERS spectra of Poly(3-hexylthiophene) in oxidized and unoxidized states. J. Raman Spectrosc. 29, 825–832 (1998).

[b31] Gao J., Thomas A. K., Johnson R., Guo H. & Grey J. K. Spatially Resolving Ordered and Disordered Conformers and Photocurrent Generation in Intercalated Conjugated Polymer/Fullerene Blend Solar Cells. Chem. Mater. 26, 4395–4404 (2014). 25678742 10.1021/cm501252y PMC4311932

[b32] Gao Y. & Grey J. K. Resonance chemical imaging of polythiophene/fullerene photovoltaic thin films: mapping morphology-dependent aggregated and unaggregated C=C Species. J. Am. Chem. Soc. 131, 9654–9662 (2009). 19601682 10.1021/ja900636z

[b33] Tsoi W. C. . The nature of in-plane skeleton Raman modes of P3HT and their correlation to the degree of molecular order in P3HT:PCBM blend thin films. J. Am. Chem. Soc. 133, 9834–9843 (2011). 21615087 10.1021/ja2013104

[b34] Yun J. J., Peet J., Cho N. S., Bazan G. C., Lee S. J. & Moskovits M. Insight into the Raman shifts and optical absorption changes upon annealing polymer/fullerene solar cells. Appl.Phys.Lett. 92, 251912–225915 (2008).

[b35] Bazzaoui E. A., Levi G., Aeiyach S., Aubard J., Marsault J. P. & Lacaze P. C. Sers Spectra of Polythiophene in Doped and Undoped States. J. Phys. Chem. 99, 6628–6634 (1995).

[b36] Bellani S. . Reversible P3HT/Oxygen Charge Transfer Complex Identification in Thin Films Exposed to Direct Contact with Water. J. Phys. Chem. C 118, 6291–6299 (2014).

[b37] Sauvajol J. L., Chenouni D., Lereporte J. P., Chorro C., Moukala B. & Petrissans J. Resonant Raman-Spectra and Photoluminescence in Polythiophene. Synth. Met. 38, 1–12 (1990).

[b38] Gao J., Thomas A. K., Johnson R., Guo H. & Grey J. K. Spatially Resolving Ordered and Disordered Conformers and Photocurrent Generation in Intercalated Conjugated Polymer/Fullerene Blend Solar Cells. Chem. Mater. 26, 4395–4404 (2014). 25678742 10.1021/cm501252y PMC4311932

[b39] Ha T. J., Sparrowe D. & Dodabalapur A. Device architectures for improved amorphous polymer semiconductor thin-film transistors. Org. Electron. 12, 1846–1851 (2011).

[b40] Pingel P., Schwarzl R. & Neher D. Effect of molecular p-doping on hole density and mobility in Poly(3-hexylthiophene). Appl.Phys.Lett. 100, 143303 (2012).

[b41] Bedogni R. . Characterization of the neutron field at the ISIS-VESUVIO facility by means of a bonner sphere spectrometer. Nucl. Instrum. Methods Phys. Res., Sect. A 612, 143–148 (2009).

[b42] Miceli A. . Measurements of gamma-ray background spectra at spallation neutron source beamlines. J. Anal. At. Spectrom. 29, 1897–1903 (2014).

[b43] Pietropaolo A. . -Ray background sources in the VESUVIO spectrometer at ISIS spallation neutron source. Nucl. Instrum. Methods Phys. Res., Sect. A 608, 121–124 (2009).

[b44] Pietropaolo A., Tardocchi M., Schooneveld E. M. & Senesi R. Characterization of the gamma background in epithermal neutron scattering measurements at pulsed neutron sources. Nucl. Instrum. Meth. A 568, 826–838 (2006).

[b45] Sears V. F. Neutron scattering lengths and cross sections. Neutron News 3, 39–37 (1992).

[b46] Street R. A., Krakaris A. & Cowan S. R. Recombination Through Different Types of Localized States in Organic Solar Cells. Adv. Funct. Mater. 22, 4608–4619 (2012).

[b47] Rabek J. F. Photodegradation of polymers: physical characteristics and applications. Springer Science & Business Media (2012).

[b48] Arthur J. C., Hinojosa O. & Tripp V. W. Effect of crystalline structure on the trapped radical spectra of irradiated cellulose. J. Appl. Polym. Sci. 13, 1497–1507 (1969).

[b49] Williams D., Schmidt B., Wolfrom M. L., Michelakis A. & McCabe L. J. Paramagnetic Resonance Spectra of Free Radicals Trapped on Irradiation of Crystalline Carbohydrates. Proc. Natl. Acad. Sci. USA 45, 1744–1751 (1959). 16590567 10.1073/pnas.45.12.1744 PMC222793

[b50] Zhao L.-H. . Role of Borderline Solvents to Induce Pronounced Extended-Chain Lamellar Order in π-Stackable Polymers. Macromolecules 44, 9692–9702 (2011).

[b51] Mayer A. C. . Bimolecular Crystals of Fullerenes in Conjugated Polymers and the Implications of Molecular Mixing for Solar Cells. Adv. Funct. Mater. 19, 1173–1179 (2009).

[b52] Andreani C. . Facility for fast neutron irradiation tests of electronics at the ISIS spallation neutron source. Appl.Phys.Lett. 92, 114101 (2008).

[b53] Platt S., Torok Z., Frost C. D. & Ansell S. Charge-Collection and Single-Event Upset Measurements at the ISIS Neutron Source. IEEE T. Nucl. Sci. 55, 2126–2132 (2008).

