# Supplementary material for: Neutron Radiation Tolerance of Two Benchmark Thiophene-Based Conjugated Polymers: the Importance of Crystallinity for Organic Avionics
Source: Sci Rep. 2017 Jan 23;7:41013. doi: 10.1038/srep41013 (PMC5253652; doi:10.1038/srep41013)

*Supplementary Information for*

**Neutron Radiation Tolerance of Two Benchmark Thiophene-Based Conjugated Polymers: the Importance of Crystallinity for Organic Avionics**

**G. M Paternò^1^, V. Robbiano^1^, K. J. Fraser^1^, C. Frost^2^, V. García Sakai^2^ and F. Cacialli^1^**

^1^London Centre for Nanotechnology, Department of Physics and Astronomy, University College London, Gower Street, London WC1E 6BT, UK

^2^ISIS Pulsed Neutron and Muon Source, Science and Technology Facilities Council, Rutherford Appleton Laboratory, Harwell Science and Innovation Campus, Didcot OX11 0QX, UK

**Table of Contents:**

**Supplementary Figure S1** Survey spectra for P3HT and PBTTT pristine, irradiated and irradiated/annealed.

**Supplementary Table ST1** XPS fitting results for pristine, irradiated and irradiated/annealed P3HT.

**Supplementary Table ST2** XPS fitting results for pristine, irradiated and irradiated/annealed PBTTT.

**Supplementary Figure S2** Non normalised UV-Vis absorption spectra for P3HT and PBTTT pristine, irradiated and irradiated/annealed.

**Supplementary Figure S3** Full Raman spectrum for P3HT (a) and PBTTT (b).

**Supplementary Figure S4** Fitting of the main in-plane ring modes of P3HT and PBTTT Raman spectra.

**Supplementary Figure S5** XRD pattern for P3HT (pristine, irradiate and irr./ann.)

**Supplementary Figure S6** OFETs characteristics for non-annealed P3HT and PBTTT devices (pristine, irradiated and irr./ann).

**Supplementary Table ST3** OFETs characteristics for non-annealed P3HT and PBTTT devices (pristine, irradiated and irr./ann).

**Supplementary Figure S1**

The mass concentrations for C (1s), O (1s) and S (2p) were calculated from the peak areas using Casa XPS software. The relative sensitivity factors (R.S.F.) used for C (1s), O (1s) and S (2p) were 1, 2.93 and 1.68, respectively. We can note a small increase of the oxygen content in both irradiated P3HT and PBTTT. However, such an increase is partially reverted by the further thermal annealing.


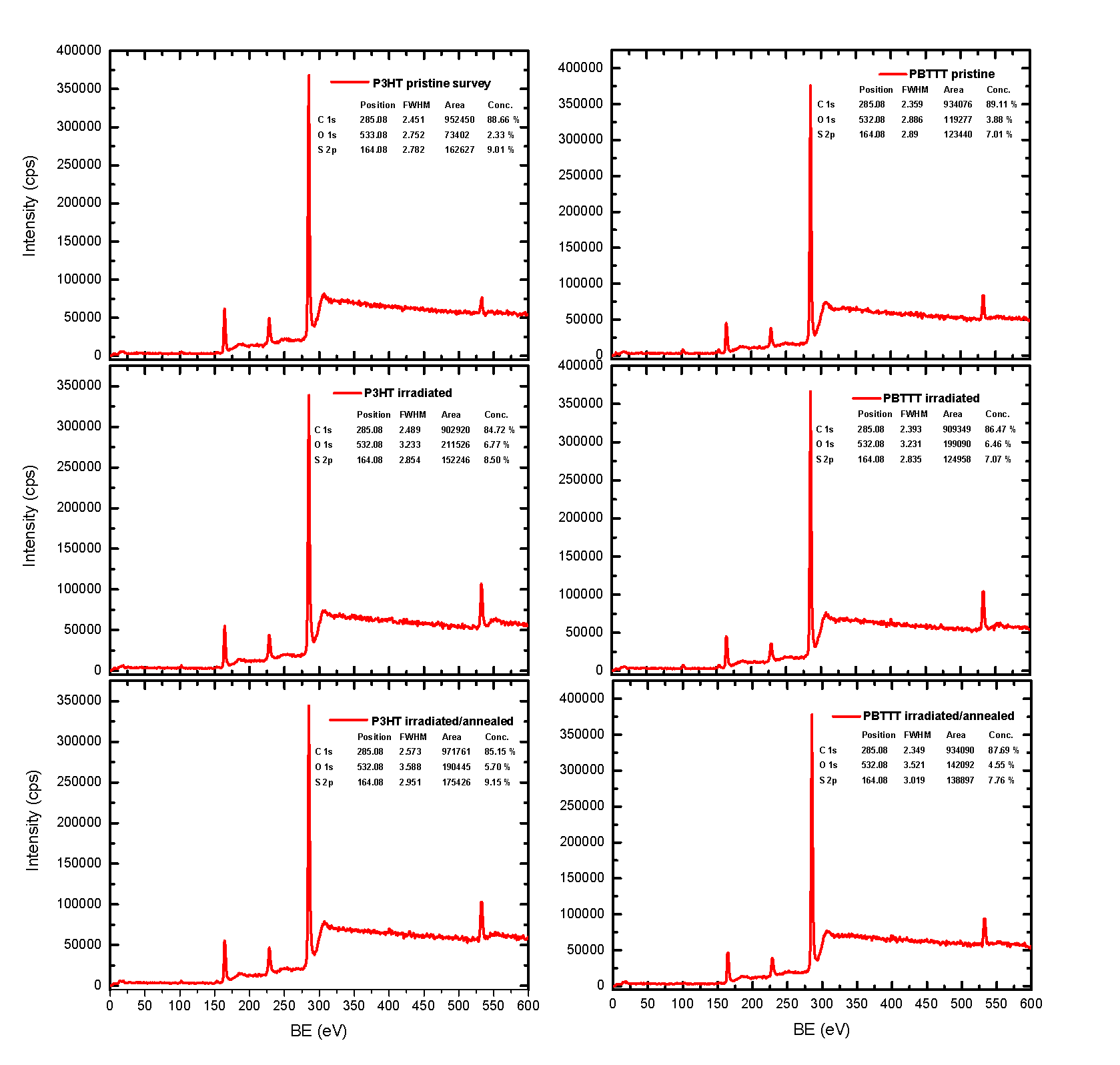


Supplementary Figure S1: Survey spectra for P3HT and PBTTT pristine, irradiated and irradiated/annealed.

Supplementary Table ST1: XPS fitting results for pristine, irradiated and irradiated/annealed P3HT.


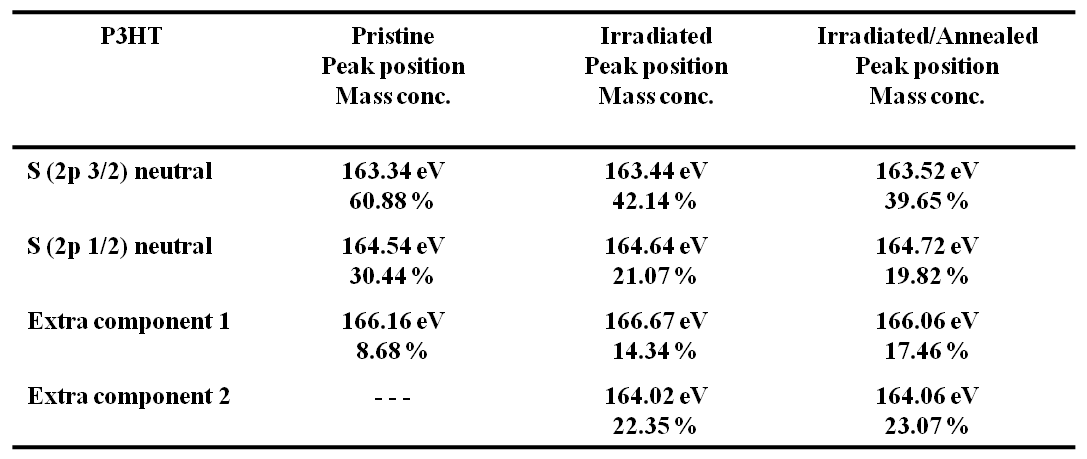


Supplementary Table ST2: XPS fitting results for pristine, irradiated and irradiated/annealed PBTTT.


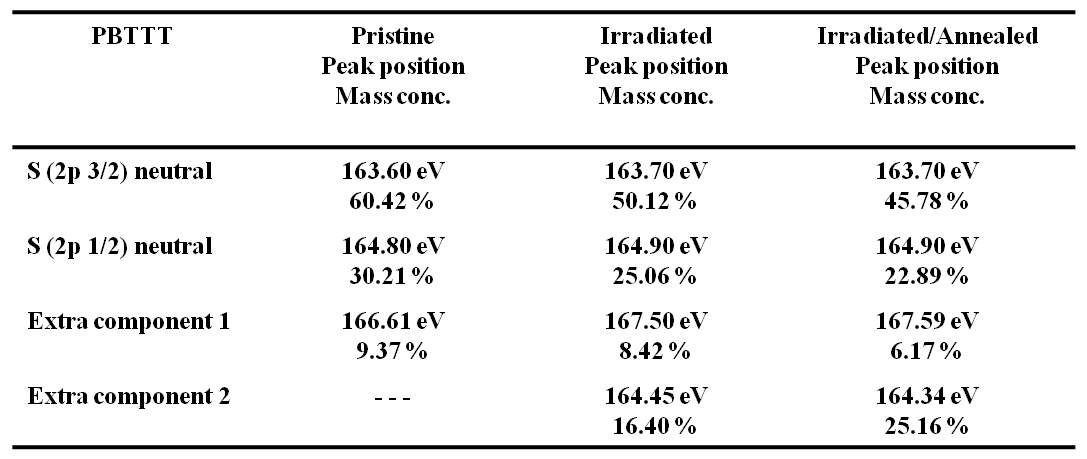


**Supplementary Figure S2**

For P3HT, the irradiation leads to a blue-shift, an increase of the sub-gap absorption and a bleaching of the absorption intensity. Interestingly, whereas the first two effects are exacerbated by the post-radiation annealing, we note that such thermal treatment permits a partial recovery of the intensity bleaching. Conversely, for PBTTT the post-irradiation annealing leads to a further bleaching of the intensity, although both the blue-shift and the sub-gap absorption are less evident than in P3HT.


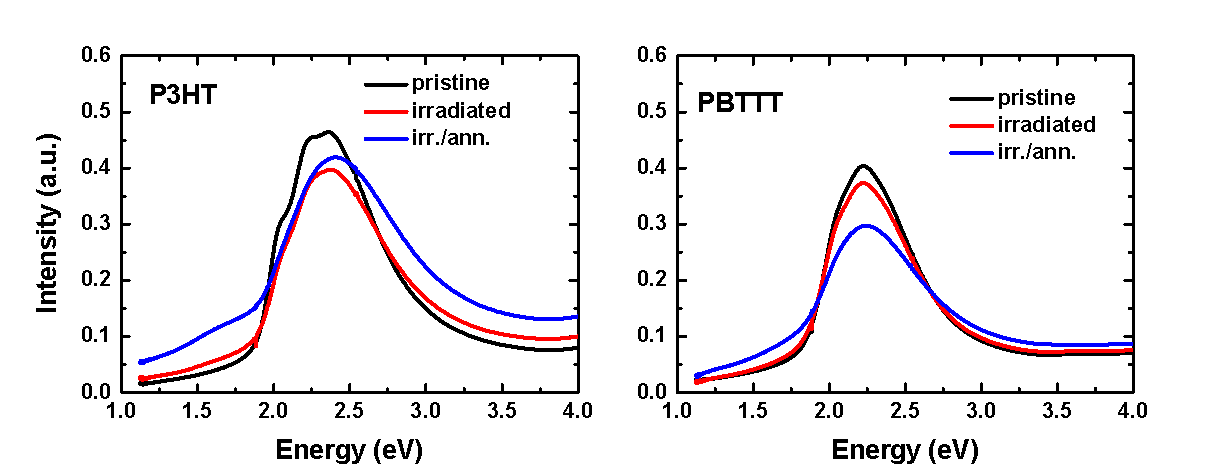


Supplementary Figure S2 UV-Vis absorption spectra for P3HT and PBTTT pristine, irradiated and irradiated/annealed.

**Supplementary Figure S3**

It is worth noting that irradiation and, to a larger extent, the post-irradiation annealing step lead to an increase in the intensity of the C-C inter-ring peak at 1210 cm^-1^, the C-H bending coupled with the C-C inter-ring mode at 1180 cm^-1^, the pure C-H bending mode at 1000 cm^-1^, the C-C_alkyl_ stretching at 876 cm^-1^ and the C-S-C ring deformation peak at 728 cm^-1^. All those effects seem to be less pronounced for PBTTT.


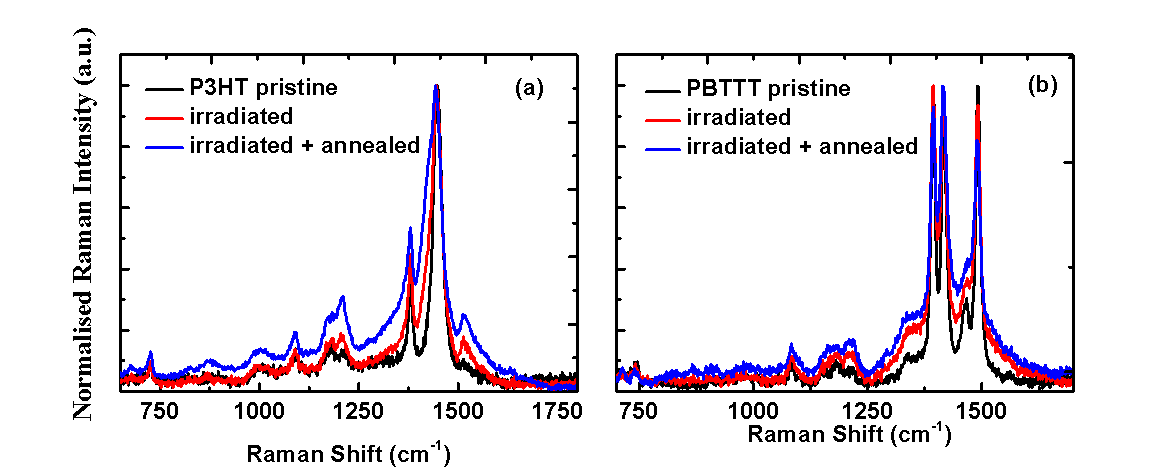


Supplementary Figure S3: Full Raman spectrum for P3HT (a) and PBTTT (b).

**Supplementary Figure S4**

For P3HT, the spectrum of pristine P3HT can be fitted using three Lorentzians, centred at 1380 cm^-1^, 1445 cm^-1^ and 1455 cm^-1^. After irradiation, we can observe the development of additional fitting components at 1350 cm^-1^, 1420 cm^-1^ and 1520 cm^-1^ that keep growing upon further annealing. The last two modes have been ascribed to the C=C stretching mode in the quinoid form and C=C asymmetric stretching, and have been related to the presence of polaronic-like species in chemically doped poly (thiophenes) [ref. 39-41]. For PBTTT, we fitted the 1300-1600 cm^-1^ region with five Lorentzians centred at 1340, 1393, 1418, 1463, and 1493 cm^-1^. Upon irradiation and post-annealing, we note a strong broadening of the C-C intra-ring mode (1420 cm^-1^) and the development of an additional fitting component at 1550 cm^-1^. This can be explained in terms of decreased conformational order after irradiation. In addition, one can note an intensity redistribution between the inter-ring C=C (1418 cm^-1^) and the intra-ring C=C (1493 cm^-1^) modes, with an increase of the latter after irradiation and annealing.


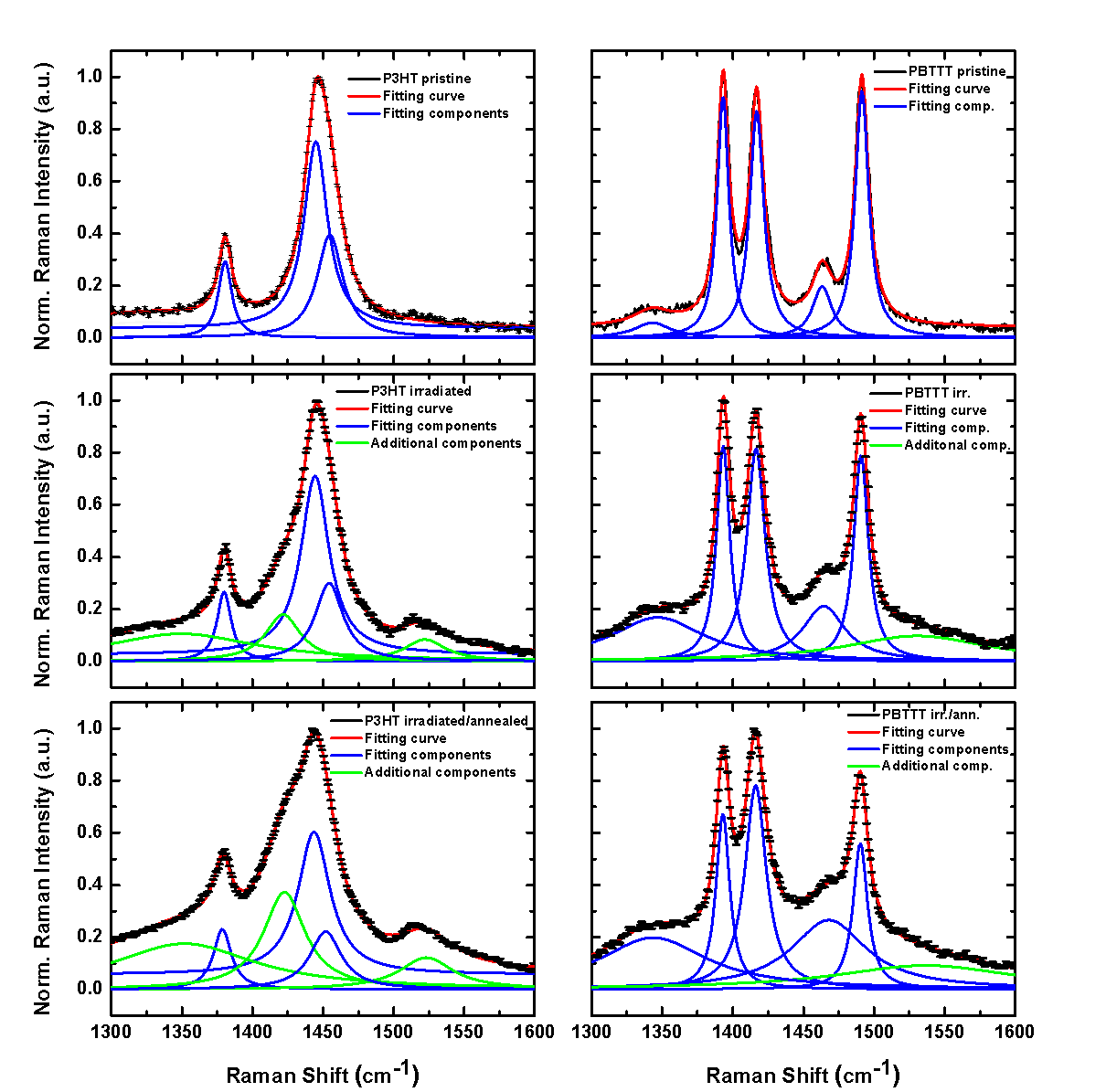


Supplementary Figure S4: Fitting of the main in-plane ring modes of P3HT and PBTTT Raman spectra.

**Supplementary Figure S5**

The XRD patterns for both P3HT and PBTTT films show no appreciable differences upon annealing and post-irradiation annealing. It is also worth noting the sharper and more intense <100> peak (lamellar stacking) observed in PBTTT, which indicates a higher crystallinity for this polymer.


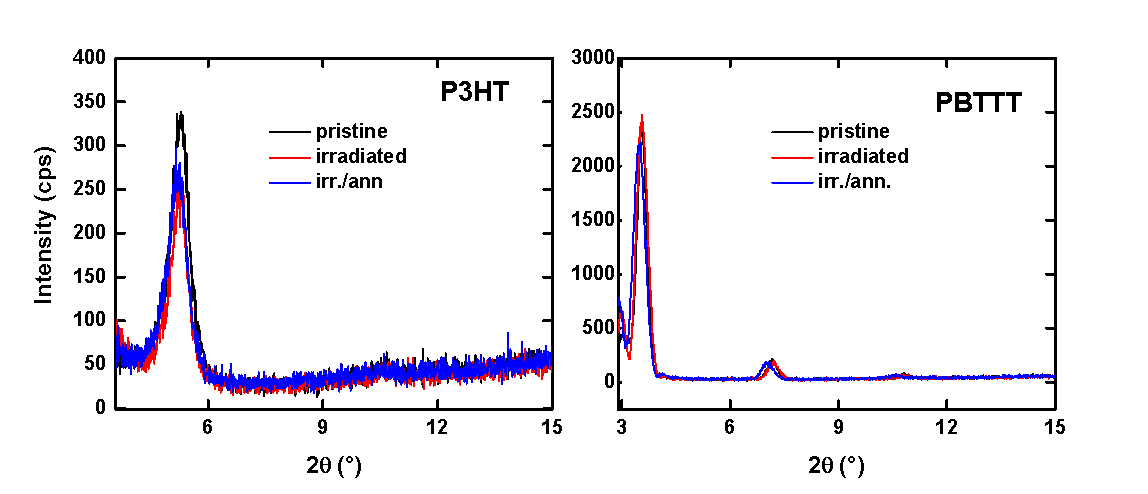


Supplementary Figure S5: XRD pattern for P3HT (left) and PBTTT (right) for pristine, irradiated and irr./ann. films.

**Supplementary Figure S6 and Supplementary Table ST3**

The OFETs characteristics for as-cast films indicate a lower radiation tolerance if the films are not thermally annealed before neutron exposure. This suggests that the crystalline phase may have a prominent role in slowing down the neutron-induced damage. In addition the hole-mobility already increases after irradiation for both the polymers, suggesting that given the lower fraction of trapped radicals in the crystalline phase, the post-irradiation annealing is not necessary in as-cast films to access a high-doping regime.


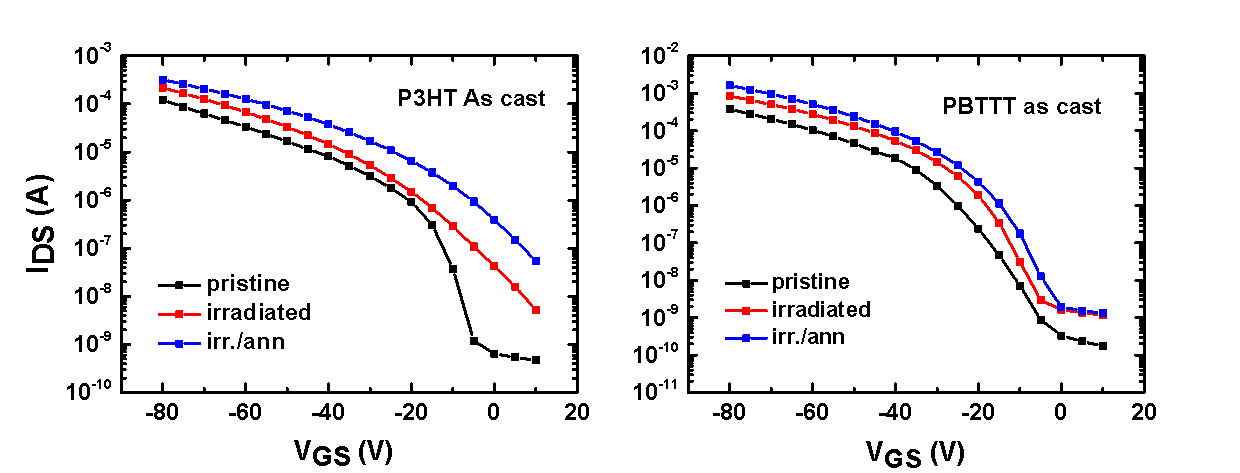


Supplementary Figure S6: OFETs characteristics for P3HT (left) and PBTTT (right) films that were not thermally annealed before the irradiation process.

Supplementary Table ST3: Hole-mobility values for P3HT and PBTTT films that were not thermally annealed before the irradiation process.


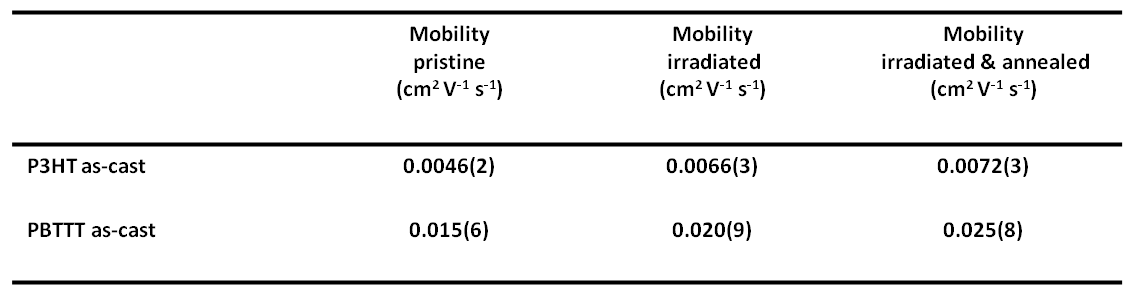

Supplement: Supplementary Information [file srep41013-s1.doc]
